# Identifying High-Cost, High-Risk Patients Using Administrative Databases in Tuscany, Italy

**DOI:** 10.1155/2017/9569348

**Published:** 2017-07-10

**Authors:** Irene Bellini, Valentina Barletta, Francesco Profili, Alessandro Bussotti, Irene Severi, Maddalena Isoldi, Maria Bimbi, Paolo Francesconi

**Affiliations:** ^1^Medical School of Hygiene and Preventive Medicine, University of Florence, Florence, Italy; ^2^Agenzia Regionale Sanità, Tuscany, Italy; ^3^Health Care Continuity Unit, University Hospital of Careggi, Florence, Italy; ^4^Local Health Authorities of Central Tuscany, Florence, Italy

## Abstract

**Objective:**

(1) Assessing the performance of the algorithm in terms of sensitivity and positive predictive value, considering General Practitioners' (GPs) judgement as benchmark, and (2) describing adverse events (hospitalisation, death, and health services' consumption) of complex patients compared to the general population.

**Data Sources:**

(i) Tuscany administrative database containing health data (2013-5); (ii) lists of complex patients indicated by GPs; and (iii) annual health registry of Tuscany.

**Study Design:**

The present study is a validation study. It compares a list of complex patients extracted through an administrative algorithm (criteria of high health consumption) to a gold standard list of patients indicated by GPs. GPs' decision was subjective but fairly well reasoned. The study compares also adverse outcomes (Emergency Room visits, hospitalisation, and death) between identified complex patients and general population.

**Principal Findings:**

Considering GPs' judgement, the algorithm showed a sensitivity of 72.8% and a positive predictive value of 64.4%. The complex cases presented here have higher incidence rates/100,000 (death 46.8; ER visits 223.2, hospitalisations 110.87, laboratory tests 1284.01, and specialist examinations 870.37) compared to the general population.

**Conclusions:**

The final validated algorithm showed acceptable sensitivity and positive predictive value.

## 1. Introduction

Several complex interventions were developed in the emerging field of multimorbidity. They are care management programmes aimed at meeting the needs of patients suffering from multiple chronic conditions, at high risk, with an important consumption of services and sited at the top of the Kaiser Permanente Pyramid [1]. These programmes are designed to assist patients and their caregivers to manage medical conditions and medical care plans, to improve the quality of care, and to reduce health care costs [2–5].

Some Italian regions are planning or testing care management initiatives to handle these problems. These projects identify patients at higher risk of hospitalisation using administrative data and subsequently improve their care at local level with multidisciplinary and multiprofessional teams. These initiatives are not coordinated with each other, and there are uncertainties regarding both the real ability to identify the most complex patients and the effectiveness of these models in providing health benefits and reducing resources' consumption.

In October 2015, a project called “Chronic Diseases: Support and Comparative Evaluation of Interventions Aimed at the Proactive Identification and Taking Charge of Complex Patients, in Order to Prevent Repetitive Hospitalisations” was approved from the Italian Ministry of Health and officially implemented in Tuscany in March 2016 [6, 7]. The first identification phase of complex cases was thus concluded. This phase has both a diagnostic and a prognostic function: complex patients were characterised through administrative data and in the meanwhile analysed for prognosis.

The objectives of this study are both development and validation of the algorithm (based on administrative data) used to identify complex patients concerning

1. performance of the model considering complex patients identified by GPs as a benchmark;
2. a comparison in terms of adverse events (hospitalisations, death, and consumption of health services) between identified complex patients and the general population.

## 2. Materials and Methods

Firstly, from December 2015 to July 2016, a literature review [8–10] was conducted in order to determine what a complex patient is and the following teamwork allowed to regard him/her as “a community dwelling patient at higher risk of repetitive visits to the Emergency Room (excluding low codes and trauma) or repetitive hospitalisations (ordinary and for medical diagnosis) for one or more chronic conditions suitable to ambulatory care. These conditions require the patient to be taken in charge by a multidisciplinary team (general practitioner, nurse, specialist, social worker, and primary health care doctor) with a personalized care plan.”

All the characteristics chosen for the definition were taken from the literature (Table 1).

For a better definition, Region Tuscany's resolution number 370 of 3/22/2010 was also considered. This regards a complete assistance for disabled patients focusing especially on complex social and health care needs. After a multidisciplinary evaluation called UVM (multidisciplinary assessment unit), since 2010 these patients have been receiving special indirect (financial support) or direct assistance (domiciliary visits by nurses or social workers or special programmes involving periodic examinations by GPs). Some of these patients can also be complex patients, but in this case they should also present the following key characteristics: chronic multimorbidity, polytherapy, and multidisciplinary health assistance needs. Complex patients should be involved in a case management programme comprehending a multidisciplinary team with specialists, more suitable to handle with complex health needs. The identification of complex patients requires an administrative algorithm and then the final judgement of the GPs. Patients at risk can be notified not just by the GPs but also by relatives, neighbours, and friends and their needs are assessed through UVM (nurse, social worker, and primary health care doctor). The consequent taking-in-charge of patients can be simpler if it involves just one type of professional (for example, nurses). Instead, complex patients having also social problems should receive social support in addition to care management and their involvement is supposed to occur according to the GP's decision.

### 2.1. Data Sources

The source of data was administrative (registry data analysis) and it protects patients privacy hiding personal data [11]. Considered databases contained the following information: hospital discharges from Tuscan public or accredited hospitals (ICD 10 CM coded diagnosis), all drugs bought, all laboratory tests, and specialist examinations.

MaCro is a database that classifies patients with chronic diseases combining administrative databases.

For each disease in the above-mentioned flows, it detects cases meeting defined criteria [12].

The yearly health registry, containing all living residents, was finally used to assess the predictivity of the algorithm and to assess the mortality rate.

#### 2.1.1. Objective  1: Performance of the Model

*List according to the Algorithm*. A list of complex patients was produced using administrative data. Complex patients were identified if they met the following criteria.

*Criterion 1*. We included community dwelling patients older than 59, whose consumption rate is in the highest 5% s, with at least one of the following characteristics:

1. More than 2 ordinary hospital admissions at medical facilities during the previous year
2. More than 2 Emergency Room (ER) visits, excluding white codes and trauma, during the previous year
3. Consumed more than 16 kinds of prescription drugs (ATC5) during the previous year
4. More than 6 laboratory tests during the previous year
5. More than 6 specialist examinations during the previous year
6. One multidisciplinary evaluation audit (composed of a community health doctor, nurse, and social worker and aimed at the drafting of a personalized care intervention) or being in a special domiciliary care programme called in Italy ADI or ADP (integrated or programmed domiciliary assistance, resp.).

*Criterion 2*. A further selection was carried out from the previous sample considering only those patients with at least one hospital admission during the previous three years for a diagnosis found in the chosen list of Aggregated Clinical Codes ACC (from now on defined as ACC database) and a revision of CCS (Clinical Classifications Software) (Table 2) or found in the MaCro database for being affected with diabetes, COPD, heart failure, ischemic heart disease, or dementia. Diagnoses with codes ICD10 CM corresponding to each ACC/CCS are available online [13].

*List of GPs*. The final list of complex patients according to GPs (gold standard) in 2015 was developed during several steps.

*First Step*. GPs produced a list of those patients among those who fulfilled the given criteria as at 12/31/2014.

The list included the following data:

- Patient identity (with fiscal code)
- Criteria for the identification of complex patients
  1. What are the patient's complex chronic conditions?
  2. Would you be surprised if the patient visited an ER and was admitted by a hospital because of a worsening of his/her chronic disease in the next 6 months?
  3. If there was an opportunity for the patient to receive proactive and multidisciplinary care, would the patient avoid ER department visits or being admitted into a hospital?
- Drugs and possible polytherapy
- Functional status and ability to move
- Cognitive status (dementia or memory disorders)
- Mental health
- Social network
- Socioeconomic status
- Living area and connections
- Life expectation (considering the surprise question: “would you be surprised if the patient died in the next 12 months?”)
- Other free considerations

51 GPs, working in 5 different Tuscan Local Health Units LHUs (which until the end of 2015 approximately corresponded to provinces) in Tuscany (Arezzo, Livorno, Massa, Lucca, and Florence), were voluntarily involved and produced the list.

*Second Step*. Fiscal codes containing all the data of each patient are currently replaced with another anonymous univocal code (called IdUni) in order to use health related data for statistical purposes without violating personal privacy [11].

Only LHUs have the key to switch from IdUni to fiscal codes and personal data of patients, so these 5 LHUs switched the fiscal codes given by GPs into IdUni codes and vice versa, in order to compare results between the lists given by the GPs and the final results of the developed algorithm. GPs received the administrative lists and could choose whether to maintain or remove cases found through the algorithm or add new ones. Validation test was conducted matching the identified complex patients through the algorithm with the lists of GPs (see further) and disaggregated according to the judgement of GPs in true positive (TP), false positive (FP), true negative (TN), and false negative (FN):

- True positives (TPs) are those patients selected both by GPs and through the algorithm.
- False positives (FPs) are those patients identified through the algorithm but not confirmed by GPs.
- False negatives (FNs) are those patients indicated by GPs but not found through the algorithm.
- True negatives (TNs) are those patients not complex for both GPs and algorithm.

Some GPs, who had previously sent their questionnaire, were removed for the following reasons:

- They did not comment on the list of administrative data.
- They had a prevalence of complex cases lower than 5%.
- They did not change their mind at least for one false positive.

The final number of GPs considered was 40.

*Final Adjustment*. FNs not detected through the algorithm and not present in the MaCro database nor in the hospitalisation registry were reanalysed considering the information given by GPs regarding active diseases with an attributable ACC code.

#### 2.1.2. Objective 2: Adverse Events' Comparison

In order to assess prognosis in terms of adverse events (hospitalisation, death, and consumption of health services) comparing identified complex patients to the general population, the final algorithm was used on the whole population living in Tuscany on 12/31/2013 and recorded in the health registry. Some indicators, such as mortality, ER visits, hospitalisations, laboratory tests, and specialist examinations, were compared with noncomplex patients in order to test the predictivity of the model. For the population at 12/31/2013 outcomes were considered during 2014 to test the capacity of the algorithm to identify patients at higher risk of adverse events in the year following the one of identification (it was chosen to study outcomes in 2014 because in 2015 considered outcomes in 2016 were not still available).

## 3. Results

**Figure 1:**
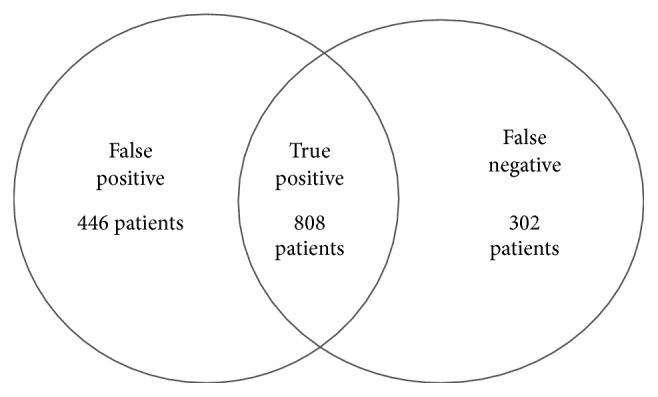
Final results.

The results of the comparison between the final administrative algorithm and the judgement of the GPs can be described in the already mentioned three groups (Figure 1):

- True positive (TP) cases were finally 808.
- False positive (FP) cases were 446, and the main reasons of exclusion explained by GPs were the following: 44%, well compensated; 25%, autonomous; and 11%, healed.
- False negative (FN) cases were 302.

Definitive complex cases were then the true positive and false negative ones.

The 40 GPs confirmed 1,110 complex cases, and the administrative algorithm found 1,254, with an initial concordance with the algorithm regarding only 260 patients, but another 506 were recognized as complex after the GPs received the lists. The administrative algorithm showed a sensitivity of 72.8% and a positive predictive value of 64.4%.

Table 3 shows that women prevailed in all three groups (TPs, FNs, and FPs), being, respectively, 52, 56, and 52%. The percentage of people aged 80 and over was higher among FNs (63%), followed by TPs (49%) and FPs (35%). Patients aged 60–74 years were more numerous among FPs (41%) than among TPs (31%) and FNs (21%). FNs were the oldest and FPs the youngest (Table 3).

Matching combined lists of complex patients found by GPs and algorithm and disaggregated in TPs, FPs, and FNs, with MaCro database (containing chronic diseases), the patients diagnosed with at least one chronic disease were 88% among TPs, 83% among FPs, and 58% among FNs. TPs always presented the highest prevalence for each disease, apart from diabetes, which is higher among FPs than TPs and FNs (33% versus 26% and 26%). FNs always showed the lowest prevalence for COPD, dementia, and diabetes but presented a higher rate of heart failure (16%) and ischemic cardiopathy (26%) compared to FPs (11 and 24%, resp.; see Table 3). Matching again the same list with ACC database, with FNs manually readjusted, the differences in the prevalence of diseases were smaller between TPs and FNs, even if TPs showed a higher burden for neurological problems (50% versus 9%). FP had a lower prevalence compared to TPs and FNs. The average number of ACC disease cases per capita was 1.1 for TPs, 0.7 for FNs, and 0.4 for FPs.

TPs had the highest health consumption too, apart for laboratory tests, performed more frequently among FP (48%) than among TPs (40%). FNs always showed the lowest health consumption (Table 3).

FNs were not detected by the algorithm for several reasons.

They had fewer diseases according to the MaCro database. 58% of FNs had at least one disease in the MaCro database, compared to 88% of TPs and 83% of FPs. Most of them were not high consumers (just 47 out of 302 met at least one consumption criterion and can be defined high consumers, as proved by the lower values in the “consumption” heading of Table 3). Among the 255 not high consumer FNs cases, 88 had at least one ACC, but it did not cause hospital admission nor any criterion for high consumption. 167 had not any ACC and they were not high consumers either. 46 were high consumers but they were not in the ACC database.

173 out of 255 FNs not high consumers were in the MaCro database with at least one diagnosis, meaning they had a chronic disease causing neither hospital admissions nor high consumption, 82 had neither MaCro diseases nor high consumption. 46 were not in the MaCro database but they were high consumers.

Matching the MaCro and hospitalisation databases for a particular ACC in the previous year, 93 patients were not found in any of these, and 25 of them turned out to be high consumers. The final adjustment we described tried to answer the question whether these FNs patients had diseases which were not considered or if they had diseases without specific related consumption. Information given by the GPs for these 93 patients was then reconsidered and manually recoded for a MaCro or ACC disease. The 25 FNs who were also high consumers but without diagnosis were also analysed to check their characteristics. For all 25 cases, we found one or more diseases, whose ACC or MaCro code could be attributed manually. The ACCs with the highest prevalence were Card1 and Neur (9 diagnoses each; see Table 2). As for the MaCro database, heart failure disease had the highest frequency (9 cases).

The FPs captured by the algorithm but rejected by the GPs were 446. The GPs reported a reason in 327 cases out of 446, and for 152 of them it was good compensation and a still autonomous lifestyle, 19 showed better conditions compared to the past exacerbations, and 12 were just following an oral anticoagulant therapy.

In the meanwhile, we studied the frequency of other characteristics found in the survey but not in the algorithm, such as functional status, social network, economic situation, area of residence, and end-of-life status on the first sample of 182 TPs patients (on a total of 808) and 300 FNs patients (out of a total of 302). These pieces of information were reported in the questionnaire filled by GPs and they were again analysed through Excel disaggregating records into TPs and FNs. The percentages were the same for TPs and FNs. For TPs, 20% were bedridden, 27% could only walk if aided, 13% had dementia, 17.5% had mental diseases, 15% lived alone, 15% were poor, 14% lived far from health care facilities, and 60% got a “No” at the surprise question.

For FNs, 13% were bedridden, 39% could only walk if aided, 13% had dementia, 18% had mental diseases, 18% lived alone, 14% were poor, 19% lived distant from health care facilities, and 60% got a “No” on the surprise question.

Overall, the prevalence of complex patients, considering our sample, is about 2.3% of the total patients found in the GPs' lists. Considering that the enrolled GPs had an average number of patients of 1,250, the average number of complex patients for each GP was then 29.

Table 4 shows the comparison for the year 2014 between the 108,479 complex cases identified in the whole population living in Tuscany as at the 12/31/2013 (population identified through the algorithm is FP + TP) and the other supposedly noncomplex patients (FN + TN). Analysing outcomes (deaths, ER visits, and hospitalisations rates) and health consumption, TP complex cases identified through the algorithm, as inferable from Table 4, presented during the year 2014 the highest incidence rates/100,000 (deaths, 46.8; ER visits, 223.2; hospitalisations, 110.87; laboratory tests, 1284.01; specialist examinations, 870.37, resp.). Noncomplex patients identified through the algorithm as TN always presented the lowest incidence rates (deaths, 37.27; ER visits, 96.9, hospitalisations, 59.63, laboratory tests, 633.57, and specialist examinations, 454.68), apart from death and hospitalisation incidence rates, which were lower among FP. FN and FP had intermediate values, excluding the two exceptions mentioned above.

## 4. Discussion

As inferable from Tables 3 and 4, the use of the algorithm is very suitable and predictive in identifying the highest costs chronic patients having a greater impact on the Health Care System and a higher probability to die because of their diseases. However, complex patients are difficult to identify because they present different characteristics. Not all people extracted through the algorithm fulfilling the given criteria to be complex are indeed true positive, according to GPs' judgement. The false positive patients identified, noncomplex, because they have lower death rates, ER visits, and hospitalisations, were anyway high consumers in terms of laboratory tests and specialistic examinations, as may be inferred from Table 4. They were detected by the algorithm because they met the criteria and had presumably more chronic diseases but were well compensated and consequently did not go to the hospital and died less often. The GPs reported indeed precisely these reasons for their exclusion. Instead, false negative patients were not extracted because they were not high consumers (laboratory tests and specialist examination rates were the lowest after TNs; see Table 4). However, they went to the hospital and died almost as often as TPs. This is in contrast with the findings reported in Table 3, where FNs seemed to present lower hospitalisation and ER visit rates. Evidently, the causes of hospitalisation were not among the ACCs chosen and consequently were not detected by the algorithm. To answer the above question, they probably had diseases that were still not codified (because they were difficult-to-reach patients) or they had codified diseases without specific related consumption (difficult-to-treat patients), but they were as ill as the TPs and with the same high risk of complications.

Other similar studies found the same difficulty [14]: counting the number of comorbid conditions does not necessarily mean that a patient is complex; instead it seemed that primary care physicians pointed that some patients of theirs with very complicated medical histories were relatively straightforward to manage (and they corresponded to the false positives often presenting a high prevalence of diseases and health consumption), whereas other patients could represent a real challenge despite relatively few medical diagnoses. Another study [15] emphasized the importance of social and behavioural contexts that can create important barriers to delivering high-quality primary care and declaring a patient to be complex and eligible for a care management programme.

Another study [16] found that PCPs considered the concept of patient complexity as a combination of medical illnesses, mental illnesses, socioeconomic challenges, and/or behaviours or traits that complicated care for chronic medical illnesses. In the same study, some physicians broadly defined complex patients as those who did not easily fit into guidelines or algorithms. The complexity is reported also as an interference with standard care and decision-making [17].

Our study did not find significant differences based on socioeconomic or social characteristics; therefore, the identification of FNs and of final complex patients is very difficult.

End-of-life patients, identified with the “surprise question,” were 60% among FNs and TPs, unexpectedly without significant differences between these two groups. However, this indicated that palliative care should be considered in the management of these patients.

The identification difficulties reflect the lack of guidelines and the difficult management of these patients. Individual experiences cannot be generalized. Another perspective is offered by a recent study [18], defining complexity as the gap between patient needs and health care services. This concept takes into account both the multiple considerations that affect the needs of patients with multiple chronic conditions and the contextual factors that influence service delivery.

The sensitivity found was 72.8%, considering GPs' judgement as a validation. However, using just the administrative algorithm is not enough; the algorithm was not perfect, as proved by FNs and FPs. Complex patients often present not detectable characteristics except through the judgement of their GPs, whose role is then essential for the validation of data and identification of FNs and FPs cases.

The main limitations of the present study were as follows: predictors were not analysed; the list considered as gold standard (GPs' judgement) was not independent from algorithm list. GPs received, indeed, the list extracted through administrative databases and were encouraged to change their mind adding or removing patients from their original lists. This unconventional method was considered the one with the highest face validity for our purpose. After several meetings among experts, being sure to “catch” all complex patients through GPs seemed the best one. GPs could indeed remember just some of these patients and the provided list extracted through the algorithm helped them to think about other patients among theirs too.

Overall, the role of administrative databases analysed by ARS was equally important, because algorithm reminded GPs, who had the task of confirming or editing patients in the list, of those high-risk cases which they had not thought of at first. They could, indeed, add those more socially vulnerable cases that, being neither big consumers nor high-costs patients, were not detected through the administrative algorithm, but who would benefit from a care management programme. In the same way, GPs could remove from their lists those cases classified as high-risk because of their high-cost but not eligible for care management.

## 5. Conclusion

Overall we can state that the final algorithm, validated through the judgement of GPs and confirmed by a further analyses on adverse outcomes, showed acceptable sensitivity and positive predictive value, even if the final lists should always be checked by the GPs because too many other behavioural, social, or different factors influence the definition and these cannot be detected with the common health administrative database.

## Figures and Tables

**Table 1:** Examples of elements of complexity found in the literature.

Dimension	Example	Sources	Number of studies
Medical/physical health	Functional impairment	[15, 19–23]	6
	Chronic symptoms	[24]	1
	Challenges in the application of clinical practice guidelines	[18, 25–29]	6
	Multimorbidity	[15, 30–44]	16
	Polypharmacy	[27, 45, 46]	3

Mental health	Mental health challenges, such as depression	[32, 47, 48]	3
	Psychological distress	[15, 32, 44, 49, 50]	5
	Cognitive impairment	[15, 51, 52]	3
	Substance use	[53, 54]	2

Social capital	Social health issues including caregiver strain	[55]	1
	Poor social support	[32, 56, 57]	5
	Relationship strain and lack of leisure time	[58]	1

Health and social experiences	Experiential challenges including poor quality of life	[32]	1
	Difficulty navigating services	[59]	1
	The need for a care manager	[60]	1
	Lack of access to providers	[57, 58]	2
	Heavy utilization of services	[28, 50, 56, 61]	4
	Higher healthcare costs	[50, 62–68]	8

Demographics	Demographic characteristics including advanced age	[15, 32, 69–72]	6
	Frailty	[21, 73, 74]	3
	Gender	[41, 44]	2
	Poverty	[15, 32, 41, 44, 75]	5
	Ethnic disparities	[76, 77]	2
	Lower level of education	[15, 32, 78]	3

Combined	One or some combination of social isolation, psychiatric illness, sociodemographic vulnerability, or other social and/or psychological difficulties	[79]	1
	Persistent distress or fear that is not adequately addressed and complicates medical management	[27]	1
	Symptom severity or impairments, diagnostic uncertainty, difficulty engaging care, lack of social safety or participation, disorganization of care, and difficult patient-clinician relationships	[16]	1

**Table 2:** Chosen list of ACC/CCS codes.

Abbreviation	ACC/CCS code	ACC category description
Card1	108′	Congestive heart failure; not hypertensive

Card2	100′	Acute myocardial infarction
	101′	Coronary atherosclerosis and other heart disease
	103′	Pulmonary heart disease
	104′	Other and ill-defined heart disease
	111′	Other and ill-defined cerebrovascular disease
	248′	Gangrene
	55′	Fluid and electrolyte disorders
	96′	Heart valve disorders
	97′	Peri-, endo-, and myocarditis; cardiomyopathy (except that caused by tuberculosis or sexually transmitted disease)
	99′	Hypertension with complications and secondary hypertension

Card3	50′	Diabetes mellitus with complications

CerVa1	109′	Acute cerebrovascular disease

CerVa2	110′	Occlusion or stenosis of precerebral arteries
	113′	Late effects of cerebrovascular disease

Gastrointestinal (GI)	6′	Hepatitis
	151′	Other liver diseases
	152′	Pancreatic disorders (not diabetes)

Cancer	11′	Cancer of head and neck
	12′	Cancer of esophagus
	13′	Cancer of stomach
	14′	Cancer of colon
	15′	Cancer of rectum and anus
	16′	Cancer of liver and intrahepatic bile duct
	17′	Cancer of pancreas
	18′	Cancer of other GI organs; peritoneum
	19′	Cancer of bronchus; lung
	20′	Cancer; other respiratory and intrathoracic
	21′	Cancer of bone and connective tissue
	23′	Other nonepithelial cancer of skin
	24′	Cancer of breast
	25′	Cancer of uterus
	26′	Cancer of cervix
	27′	Cancer of ovary
	28′	Cancer of other female genital organs
	29′	Cancer of prostate
	30′	Cancer of testis
	31′	Cancer of other male genital organs
	32′	Cancer of bladder
	33′	Cancer of kidney and renal pelvis
	34′	Cancer of other urinary organs
	35′	Cancer of brain and nervous system
	36′	Cancer of thyroid
	37′	Hodgkin's disease
	38′	Non-Hodgkin's lymphoma
	39′	Leukemias
	40′	Multiple myeloma
	41′	Cancer; other and unspecified primary
	42′	Secondary malignancies
	43′	Malignant neoplasm without specification of site
	44′	Neoplasms of unspecified nature or uncertain behaviour

Kidn	156′	Nephritis; nephrosis; renal sclerosis
	158′	Chronic kidney disease

Neur	227′	Spinal cord injury
	653′	Delirium dementia and amnestic and other cognitive disorders
	79′	Parkinson's disease
	80′	Multiple sclerosis
	85′	Coma; stupor; and brain damage

Resp	122′	Pneumonia (except that caused by tuberculosis or sexually transmitted disease)
	127′	Chronic obstructive pulmonary disease and bronchiectasis
	128′	Asthma
	129′	Aspiration pneumonitis; food/vomitus
	131′	Respiratory failure; insufficiency; arrest (adult)

**Table 3:** Characteristics and outcomes in 2015 for complex patients disaggregated by TP, FN, and FP.

True positive			False negative			False positive		
Age group	Number	Percentage	Age group	Number	Percentage	Age group	Number	Percentage
60–74	251	31	60–74	63	21	60–74	181	41
75–79	162	20	75–79	50	17	75–79	108	24
80+	395	49	80+	189	63	80+	157	35

Total	808	100	Total	302	100	Total	446	100

Gender	Number	Percentage	Gender	Number	Percentage	Gender	Number	Percentage

Male	389	48	Male	133	44	Male	216	48
Female	419	52	Female	169	56	Female	230	52

Total	808	100	Total	302	100	Total	446	100

Number and prevalence (%) considering MaCro			Number and prevalence (%) considering MaCro			Number and prevalence (%) considering MaCro		
Chronic diseases	Number	Percentage	Chronic diseases	Number	Percentage	Chronic diseases	Number	Percentage

Heart failure	170	21	Heart failure	48	16	Heart failure	47	11
Ischaemic cardiopathy	284	35	Ischaemic cardiopathy	80	26	Ischaemic cardiopathy	107	24
COPD	282	35	COPD	64	21	COPD	145	33
Dementia	110	14	Dementia	16	5	Dementia	34	8
Diabetes	211	26	Diabetes	78	26	Diabetes	147	33
At least one chronic disease	713	88	At least one chronic disease	174	58	At least one chronic disease	368	83

Number and prevalence (%) considering hospitalisation database			Number and prevalence (%) considering hospitalisation database			Number and prevalence (%) considering hospitalisation database		
ACC	Number	Percentage	ACC	Number	Percentage	ACC	Number	Percentage

Card 1	74	9	Card 1	29	10	Card 1	13	3
Card 2	187	23	Card 2	66	22	Card 2	81	18
Card3	10	1	Card3	6	2	Card3	0	0
CerVa1	49	6	CerVa1	31	10	CerVa1	19	4
CerVa2	7	1	CerVa2	2	1	CerVa2	2	0
GI	8	1	GI	7	2	GI	5	1
Canc	30	4	Canc	27	9	Canc	16	4
Kidn	13	1	Kidn	5	2	Kidn	7	2
Neur	401	50	Neur	27	9	Neur	3	1
Resp	116	14	Resp	37	12	Resp	30	7
At least one	401	50	At least one	153	51	At least one	159	36

*Average number of diseases*	1.1		*Average number of diseases*	0.7		*Average number of diseases*	0.4	

Consumptions	Number	Percentage	Consumptions	Number	Percentage	Consumptions	Number	Percentage

Hospital admissions	101	13	Hospital admissions	1	0	Hospital admissions	30	7
Average number of hospitalisations	399/808 = 4.5		Average number of hospitalisations	51/302 = 0.16		Average number of hospitalisations	139/446 = 0.31	
ER admissions	205	25	ER admissions	13	4	ER admissions	98	22
Examinations	198	25	Examinations	16	5	Examinations	109	24
Laboratory tests	320	40	Laboratory tests	21	7	Laboratory tests	214	48
ATC	309	38	ATC	13	4	ATC	67	15
UVM/ADP	163	20	UVM/ADP	10	3	UVM/ADP	70	16

**Table 4:** Rates/100 person-years in 2014.

	Death rate		ER visits (no trauma)		Hospitalisation (medical area)		Laboratory tests (prescriptions)		Specialist examinations	
	IR	IRR	IR	IRR	IR	IRR	IR	IRR	IR	IRR
FP	8.06	1	135.77	1	52.07	1	1226.85	1	685.03	1

TN	37.27	4.62 (1.65–12.97) *P*: 0.004	96.9	0.71 (0.41–1.25) *P*: 0.237	59.63	1.14 (0.55–2.36) *P*: 0.714	633.57	0.52 (0.42–0.64) *P*: 0.000	454.68	0.66 (0.51–0.86) *P*: 0.002

FN	38.3	4.75 (2.54–8.88) *P*: 0.000	200.1	1.47 (1.22–1.78) *P*: 0.000	106.28	2.04 (1.54–2.71) *P*: 0.000	707.54	0.58 (0.53–0.63) *P*: 0.000	586.91	0.86 (0.78–0.95) *P*: 0.002

TP	46.78	5.80 (3.28–10.27) *P*: 0.000	223.21	1.64 (1.41–1.92) *P*: 0.000	110.87	2.13 (1.67–2.71) *P*: 0.000	1284.01	1.05 (0.99–1.10) *P*: 0.106	870.37	1.27 (1.18–1.36) *P*: 0.000
